# Ratiometric ultrasensitive electrochemical immunosensor based on redox substrate and immunoprobe

**DOI:** 10.1038/srep35440

**Published:** 2016-10-14

**Authors:** Zhongxue Tang, Zhanfang Ma

**Affiliations:** 1 Department of Chemistry, Capital Normal University, Beijing 100048, China

## Abstract

In this work, we presented a ratiometric electrochemical immunosensor based on redox substrate and immunoprobe. Carboxymethyl cellulose-Au-Pb^2+^ (CMC-Au-Pb^2+^) and carbon-Au-Cu^2+^ (C-Au-Cu^2+^) nanocomposites were firstly synthesized and implemented as redox substrate and immunoprobe with strong current signals at −0.45 V and 0.15 V, respectively. Human immunoglobulin G (IgG) was used as a model analyte to examine the analytical performance of the proposed method. The current signals of CMC-Au-Pb^2+^ (I_substrate_) and C-Au-Cu^2+^ (I_probe_) were monitored. The effect of redox substrate and immunoprobe behaved as a better linear relationship between I_probe_/I_substrate_ and Lg C_IgG_ (ng mL^−1^). By measuring the signal ratio I_probe_/I_substrate_, the sandwich immunosensor for IgG exhibited a wide linear range from 1 fg mL^−1^ to 100 ng mL^−1^, which was two orders of magnitude higher than other previous works. The limit of detection reached 0.26 fg mL^−1^. Furthermore, for human serum samples, the results from this method were consistent with those of the enzyme linked immunosorbent assay (ELISA), demonstrating that the proposed immunoassay was of great potential in clinical diagnosis.

Electrochemical immunoassay has become an effective analytical method for the determination of ultra-trace antibodies and antigens, owing to its advantages of high sensitivity, low cost, rapid detection, miniaturization^1^. For sensitive detection of ultra-trace target, signal amplification procedure is necessary, which is commonly realized by using nanomaterials and enzymes to promote the electron transport^2^,^3^,^4^. Generally, the current response of amperometric immunoassay is at tens of nanoamperes or several microamperes levels^5^,^6^, which makes the assay become vulnerable to surroundings, resulting in an issue of the reproducibility of electrochemical immunoassay.

The two-channel ratiometric detection has been introduced in fluorescence^7^,^8^, colorimetric^9^,^10^ and electrochemical analysis^10^,^11^, recording two parallel signals at different wavelengths or redox potentials. Compared to measurements performed with single signal, two-channel ratiometric strategy has been shown to increase accuracy and reproducibility of targets detection^12^,^13^. Up to now, ratiometric electrochemical detections of ultra-trace heavy metal ions^10^, small molecules^14^ and DNA^15^,^16^, which is commonly assisted by oligonucleotide, have attracted many attentions. Ratiometric detection based on two-channel method is promising for realizing ultrasensitive and highly reproducible electrochemical immunoassay.

Recently, some DNA-assisted two-channel ratiometric electrochemical immunoassays have been reported^17^,^18^. In these methods, DNA hybridization was employed, using the redox species-labeled oligonucleotides to functionalize antibodies and substrate, to monitor the immunoreaction. Although the DNA-assisted ratiometric electrochemical immunoassay exhibited good reproducibility and sensitivity, the limitation of this approach was that the complex, expensive and time-consuming preparation of the redox species-labeled oligonucleotides and the oligonucleotide-tagged antibodies was involved. If these obstacles were resolved by DNA-free method, the two-channel ratiometric immunoassay will be of great significance in clinical diagnosis.

Herein, a novel DNA-free ratiometric sandwich electrochemical immunoassay was firstly developed using redox substrate and immunoprobe. Carboxymethyl cellulose-Au-Pb^2+^ (CMC-Au-Pb^2+^) nanocomposites and carbon-Au-Cu^2+^ (C-Au-Cu^2+^) nanocomposites, as two new redox materials with powerful current signals at −0.45 V and 0.15 V, were synthesized by electrostatic adsorption and coordination effect, respectively. CMC-Au-Pb^2+^ and C-Au-Cu^2+^ were implemented as redox substrate and probe, respectively. The human immunoglobulin G (IgG) served as a model analyte^6^. By measuring the ratio of peak currents of immunoprobe and substrate (I_probe_/I_substrate_), the proposed immunosensor for IgG detection exhibited a wide linear detection range from 1 fg mL^−1^ to 100 ng mL^−1^, which was two orders of magnitude wider than other sandwich immunoassays. For human serum detection, the results of the proposed method were consistent with those of ELISA method, demonstrating its great potential in clinical diagnosis.

## Results and Discussion

**Figure 1:**
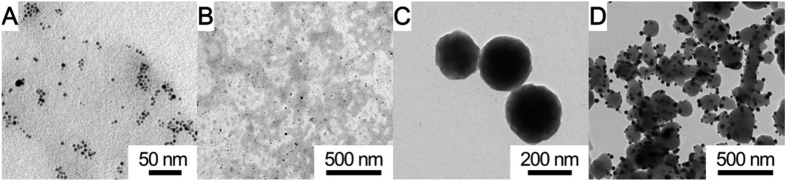
TEM images of CMC-Au (**A**,**B**), CNP (**C**) and CNP-Au (**D**).

Transmission electron microscopy (TEM) images revealed that Au nanoparticle (AuNP) about 10 nm in diameter was densely distributed on the as-synthesized carboxymethyl cellulose-Au (CMC-Au) film (Fig. 1A,B and S1A). Scanning electron microscopy (SEM) images indicated that an even CMC-Au film turned out to be rough after coating Pb^2+^ (Fig. S1B). The obtained CMC-Au-Pb^2+^ film was insoluble and could firmly adhere to the electrode, resulting from the chelating properties between CMC and lead ions^19^,^20^.

After the stepwise microwave-assisted reactions, the carbon nanoparticle (CNP) approximately 200 nm in size was obtained (Fig. 1C), and AuNPs about 30 nm in diameter were distributed on the carbon nanoparticle-Au (CNP-Au) nanocomposites (Fig. 1D). X ray photoelectron spectra (XPS) were performed to investigate the composition of CMC-Au-Pb^2+^ and C-Au-Cu^2+^ (Fig. S2). C1s and O1s corresponded to CMC (Fig. S2A) and CNP (Fig. S2D). The Au4f peaks (83.9 eV and 87.5 eV; 84.4 eV and 87.6 eV) were consistent with Au^0^,^21^ demonstrating the existence of AuNP (Fig. S2B,E). For CMC-Au-Pb^2+^, the Pb4f peaks (139.1 eV and 143.8 eV) were consistent with Pb^2+^^22^, indicating that Pb^2+^ was successfully adsorbed by CMC-Au (Fig. S2A,C). While the Cu2p peaks (935.9 eV, 943.4 eV, 955.5 eV and 963.3 eV) consistent with Cu^2+^ proved that Cu^2+^ was adsorbed by CNP-Au (Fig. S2D,F)^23^.

CMC-Au and CNP-Au were characterized by UV-visible (UV-vis) spectra (Fig. S3). No adsorption peaks were observed for CMC and CNP. After the reduction of HAuCl_4_, two peaks at 516 nm and 536 nm were observed for CMC-Au and CNP-Au, respectively. These adsorption peaks were mainly derived from Au nanoparticles, suggesting that CMC-Au and CNP-Au were obtained, and their peak position differences were mainly caused by the size of Au nanoparticles^3^. After CNP-Au was mixed with Cu^2+^, the adsorption peak shifted to 536 nm. Furthermore, CMC-Au-Pb^2+^ and CNP-Au-Cu^2+^ were characterized by Fourier transform infrared spectra (FT-IR) (Fig. S4A,B). The peaks at 2980 cm^−1^ corresponded to methylene and methyl groups. The peaks at 1384 cm^−1^ and 1639 cm^−1^ were from methyl groups and carbonyl groups, respectively. No specific peaks were observed for CNP at <1500 cm^−1^, resulting from the carbonation of glucose. The peaks at 3500 cm^−1^ were derived from carboxyl and hydroxyl groups.

**Figure 2:**
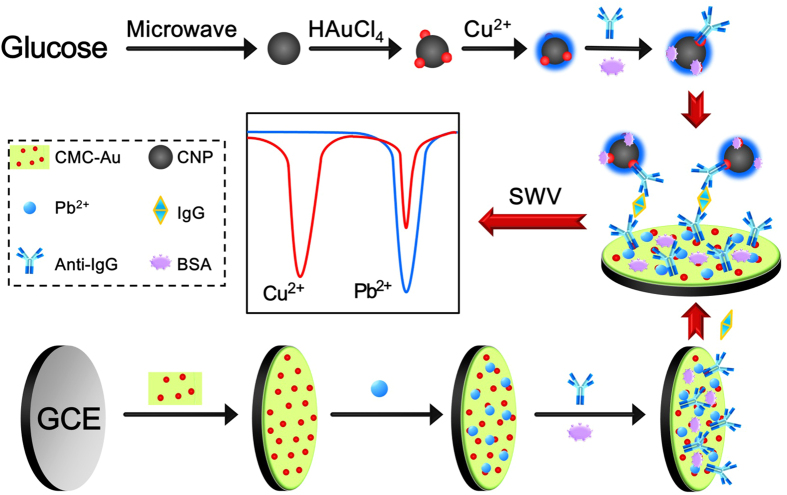
Schematic illustration of the function principle and the fabrication procedure of the proposed immunosensor.

Conventionally, in sandwich immunoassay, the capture antibodies and labeled antibodies were immobilized on the substrate and the probes, respectively. Antigens were subsequently recognized by the substrate and probe^24^,^25^,^26^. If the substrate was fabricated by redox material^27^,^28^, the analyte can be detected simultaneously by the signals of substrate and probe, respectively. In this case, the DNA-free ratiometric immunoassay was presented and its principle was illustrated in Fig. 2. CMC-Au-Pb^2+^ and C-Au-Cu^2+^ were simply synthesized by mixing CMC-Au with Pb^2+^ and CNP-Au with Cu^2+^, which were used as redox substrate and probes, respectively. After immunoreaction and electrochemical measurement, two parallel signals of Cu^2+^ and Pb^2+^ were obtained. Based on the linear relation between I_probe_/I_substrate_ and Lg C_IgG_ (ng mL^−1^), the quantity of IgG was determined.

The cross-talk level of redox peaks of CMC-Au-Pb^2+^ and C-Au-Cu^2+^ was investigated by square wave voltammetry (SWV) (Fig. S5). No current peaks for CMC-Au and CNP-Au appeared in the potential range of −1.3 V to 0.5 V. In contrast, two strong signals at −0.45 V and 0.15 V for CMC-Au-Pb^2+^ and C-Au-Cu^2+^ were observed, which were derived from the enriched Pb^2+^ and Cu^2+^. In this case, CMC-Au-Pb^2+^ and C-Au-Cu^2+^ were used as the redox substrate and immunoprobe.

**Figure 3:**
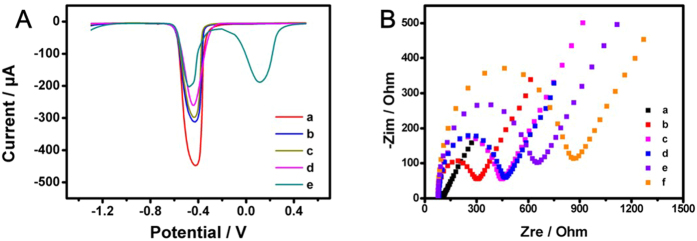
(**A**) SWV responses of the modified electrodes: CMC-Au-Pb^2+^/GCE (a), Anti-IgG/CMC-Au-Pb^2+^/GCE (b), BSA/Anti-IgG/CMC-Au-Pb^2+^/GCE (c), IgG/BSA/Anti-IgG/CMC-Au-Pb^2+^/GCE (d) and C-Au-Cu^2+^-IgG/IgG/BSA/Anti-IgG/CMC-Au-Pb^2+^/GCE (e). (**B**) EIS of the modified electrodes: bare GCE (a), CMC-Au-Pb^2+^/GCE (b), Anti-IgG/CMC-Au-Pb^2+^/GCE (c), BSA/Anti-IgG/CMC-Au-Pb^2+^/GCE (d), BSA/Anti-IgG/CMC-Au-Pb^2+^/GCE (e) and C-Au-Cu^2+^-IgG/BSA/Anti-IgG/CMC-Au-Pb^2+^/GCE (f).

The stepwise fabrication procedures of this immunosensor were monitored by SWV (Fig. 3A) and electrochemical impedance spectroscopy (EIS) (Fig. 3B), respectively. After Pb^2+^ incubated on the CMC-Au modified glassy carbon electrode (GCE), a strong current peak at −0.45 V was observed, suggesting that Pb^2+^ was successfully adsorbed by CMC-Au (curve a). The current signal of substrate gradually decreased when goat-anti-human immunoglobulin G (anti-IgG), bovine albumin (BSA) and IgG were subsequently incubated on the substrate (curve b-d), indicated that the immunosensor was obtained and IgG was recognized. The signal decrease was caused by anti-IgG, BSA and IgG, which can hinder the electron transport^29^. After the incubation of immunoprobe, the signal decrease of substrate was observed and the signal of probe appeared (curve e). EIS was performed in 5 mM [Fe(CN)_6_]^4−/3−^ aqueous solution containing 0.1 M KCl. The Nyquist plots were shown in Fig. 3B. Compared with bare GCE (curve a), a larger semicircle was obtained for electrode covered with CMC-Au-Pb^2+^ film (curve b). The diameter of semicircle gradually increased when anti-IgG and BSA were subsequently coated, suggesting that the immunosensor was obtained (curve c and d). Then the resistance increased with the incubation of IgG, indicating that IgG immune reacted with anti-IgG (curve e). The resistance further increased when the probes were coated, demonstrating that the probe was fixed (curve f). These resistance increases were derived from that the proteins hindered the electron transport^30^,^31^.

The pH of the detection solution can significantly affect the sensitivity of the immunosensor. The effect of pH on the current response was investigated as shown in Fig. S6. After immunoassay, the I_substrate_ generally increased from pH 4.0 to 5.5 and then slightly decreased from pH 5.5 to 6.0. The change trend of I_probe_ caused by pH was similar with that of I_substrate_. This indicated that pH 5.5 was optimal for detecting IgG.

**Figure 4:**
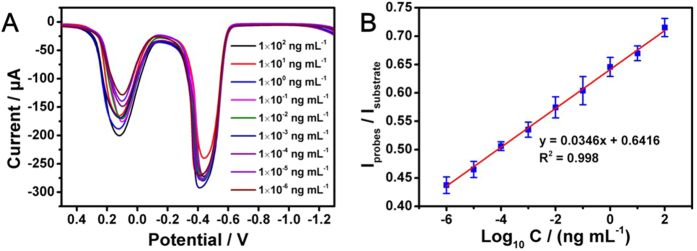
(**A**) SWV responses of the immunosensor for different concentrations of IgG. (**B**) Calibration curves between the I_substrate_/I_probe_ and logarithm of IgG concentration.

Under the optimized pH, the analytical property of the proposed immunosensors for IgG was investigated by detecting a series of standard IgG samples (Fig. 4A). I_substrate_ was irregularly changed with adding IgG, which was consistent with that of I_probe_. Obviously, no satisfactory calibration curves would be obtained between single current signal (I_substrate_ or I_probe_) and logarithm of IgG concentration. Unexpectedly, a good linear relation between I_probe_/I_substrate_ and logarithm of IgG concentration existed (Fig. 4B). The linear regression equation was I_probe_/I_substrate_ = 0.0346Lg C (ng mL^−1^) + 0.6416 with a correlation coefficient of 0.998. The proposed immunosensor for IgG exhibited a wide linear detection range (LDR) from 1 fg mL^−1^ to 100 ng mL^−1^ with an ultralow limit of detection (LOD) of 0.26 fg mL^−1^ (Fig. 4B). The results indicated that the two-channel ratiometric method was effective for ultrasensitive electrochemical immunoassay. The comparison of this method with some previous works was illustrated in the Table 1. It can be seen that present method possessed a wider LDR and lower LOD.

To investigate the repeatability, 10 ng mL^−1 ^IgG was determined by the proposed immunosensor for three times. The results showed small deviation of ±2.35%, proving its fantastic repeatability. In order to testify the specificity, the 10 ng mL^−1 ^IgG samples containing 100 ng mL^−1^ interfering substances, such as glucose (GC), dopamine (DA), uric acid (UA), ascorbic acid (AA), glutamic acid (Glu), lactic acid (LA) and BSA, were analyzed. As shown in Fig. S7, the obtained current signals were not affected by the interference substances, demonstrating the admirable specificity of this immunosensor. The stability of the immunosensor was examined by determining the IgG. After 14 days, the capacity of the immunosensor remained 85%, suggesting the good stability of the immunosensor. Compared with some recently reports, results indicated that the proposed immunosensor was more sensitive (Table 1).

Generally, for a normal adult, the concentration of IgG is about 7–16 mg mL^−1^ in serum. The human serum was firstly determined by ELISA method. After being diluted 1:10^6^, the received human serum samples were investigated by the proposed immunosensor. The results were listed in the Table S1, and relative error (RE) was less than 10%. The results of this immunoassay were consistent with the ELISA method, indicating its great potentials in clinic diagnosis.

## Conclusion

In summary, CMC-Au-Pb^2+^ and C-Au-Cu^2+^ firstly synthesized as redox species, with parallel current signals. A novel ratiometric electrochemical immunosensor was successfully developed using CMC-Au-Pb^2+^ and C-Au-Cu^2+^ to construct the redox substrate and probe, respectively. The linear range of the proposed immunoassay (1 fg mL^−1^ to 100 ng mL^−1^), two orders of magnitude higher than other sandwich electrochemical immunoassay. This immunosensor exhibited excellent sensitivity, reproducibility, specificity, stability and practicability. This ratiometric method could be easily extended to the detection of other targets.

## Methods

### Materials

CMC (M.W. 250000) was purchased from Aladdin Industrial Corporation (Shanghai, China). Pb(NO_3_)_2_ and Cu(NO_3_)_2_·3H_2_O were bought from Sinopharm Chemical Reagents Co., Ltd. (Beijing, China). AA (99.7%), UA, HAuCl_4_·3H_2_O (99.9%), GC, LA, Glu and dopamine hydrochloride were commercially obtained from Alfa Aesar (Beijing, China). IgG, anti-IgG and BSA (standard grade) were obtained from Xinjingke Biotechnology Co., Ltd. (Beijing, China). Clinical human serum samples were provided by Capital Normal University Hospital (Beijing, China). All other reagents were used as received. Deionized-distilled water was purified by Olst ultrapure K8 apparatus (Olst, Ltd., resistivity > 18 MΩ cm).

### Apparatus

TEM was performed with a JEOL-100CX electron microscope under 80 kV accelerating voltage (H7650, Hitachi, Japan). SEM was performed with a Hitachi SU8010 SEM. X-ray photoelectron spectroscopy (XPS) analysis was conducted on an ESCALAB 250 X-ray Photoelectron Spectroscope (Thermofisher, American). The UV-vis spectroscopy and Fourier transform infrared spectroscopy (FT-IR) were recorded by a UV-2550 spectrophotometer (Shimadzu, Japan) and a FT-IR spectrophotometer, respectively. EIS measurements were performed using Princeton PARSTAT 2273 (America). SWV was performed on a CHI832 electrochemical work station (Chenhua Instruments Co., Shanghai, China) with a three-electrode system which was consisted of a platinum wire as the auxiliary electrode, an Ag/AgCl electrode (saturated KCl) as the reference electrode and a GCE as the working electrode.

### Synthesis of C-Au-Cu^2+^ immunoprobe

The stepwise fabrication procedures of immunoprobe were shown in Fig. 2. CNP was synthesized according to the literature^32^. In brief, a homogeneous solution was produced by 1% glucose mixed with 10% sodium citrate. Further, the prepared solution was heated to 170 °C for 30 min in microwave reaction instrument (250 W). CNP was centrifuged, dispersed. After mixing CNP with 100 μL 4% HAuCl_4_, a microwave reaction (100 °C, 10 min) was carried out. The CNP-Au nanocomposite was centrifuged and dispersed to 5 mL water. For adsorbing Cu^2+^, 1 mL CNP-Au was mixed with 1 mL100 mM Cu^2+^ for 3 h. Then the obtained C-Au-Cu^2+^ was centrifuged, washed and dispersed to 1 mL water. 5 mL C-Au-Cu^2+^ was mixed with 500 μL anti-IgG with stirring gently for 8 h. After that, the obtained C-Au-Cu^2+^/anti-IgG was blocked by 1% BSA for 1 h, and CNP-Au-Cu^2+^/anti-IgG/BSA was centrifuged, washed for several times, and dispersed to 5 mL water.

### Synthesis of CMC-Au

After a homogeneous solution was prepared by 1 mL 1% CMC and 10 μL 4% HAuCl_4_, 40 μL 1.5% NaBH_4_ was quickly injected with vortex mixing for 1 min. The solution turned from light yellow to red. Then, the CMC-Au was obtained and diluted to 16 mL prior to use.

### Fabrication of immunosensor

The schematic illustration of the fabrication procedure of the proposed immunosensor was shown in Fig. 2. GCE (Φ = 4 mm) was prepared by polishing with the alumina powders and successively sonication washing with water. After 20 μL CMC-Au was dropped and dried at 37 °C, the obtained GCE was dipped in 10 mM Pb^2+^ for 10 min. After that, a thin CMC-Au-Pb^2+^ film was formed and washed with plenty of deionized water. 80 μL 100 μg mL^−1^ anti-IgG was incubated on CMC-Au-Pb^2+^/GCE for 12 h at 4 °C. The obtained electrode was incubated with 1% BSA at 37 °C for 1 h to block the remaining active points. Therefore, the immunosensor was accomplished and stored at 4 °C.

### Electrochemical measurement

The immunosensor was incubated with 80 μL IgG for 1 h at 37 °C. Next, 20 μL C-Au-Cu^2+^/anti-IgG/BSA was incubated for 1 h at 37 °C. The modified GCE was carefully washed with water after each step. Subsequently, SWV was performed from −1.3 V to 0.6 V in acetate buffer with pulse amplitude of 25 mV and an increase E of 4 mV s^−1^.

## Additional Information

**How to cite this article**: Tang, Z. and Ma, Z. Ratiometric ultrasensitive electrochemical immunosensor based on redox substrate and immunoprobe. *Sci. Rep.* **6**, 35440; doi: 10.1038/srep35440 (2016).

## Supplementary Material

Supplementary Information

## Figures and Tables

**Table 1:** Comparison of the performances of the present and referenced electrochemical biosensors for IgG.

Method*	LDR	LOD	Refs
	(ng mL^−1^)	(ng mL^−1^)	
DPV	0.01–10.0	6.9 × 10^−3^	33
DPV	0.1–1 × 10^5^	0.05	34
DPV	0.01–500	9.7 × 10^−3^	35
DPV	0.01–100	1 × 10^−3^	36
EIS	0.5–125	0.02	37
i-t	5 × 10^−5^–5	3.2 × 10^−6^	38
LSV	1 × 10^−6^–1	5 × 10^−7^	39
PC	1 × 10^−5^–1.0	6 × 10^−6^	40
SWV	30-1 × 10^3^	25	41
SWV	1 × 10^−3^–0.1	5 × 10^−5^	42
SWV	1 × 10^−5^–100	2.6 × 10^−6^	This Work

^*^Differential pulse voltammetry (DPV), amperometric i-t curve (i-t), photocurrent analysis (PC), linear sweep voltammetry (LSV).
