# Supplementary material for: Ratiometric ultrasensitive electrochemical immunosensor based on redox substrate and immunoprobe
Source: Sci Rep. 2016 Oct 14;6:35440. doi: 10.1038/srep35440 (PMC5064308; doi:10.1038/srep35440)
Supplement: Supplementary Information [file srep35440-s1.doc]

Supplementary material

**Ratiometric ultrasensitive electrochemical immunosensor based on redox substrate and immunoprobe**

Zhongxue Tang, Zhanfang Ma*

Department of Chemistry, Capital Normal University, Beijing 100048, China


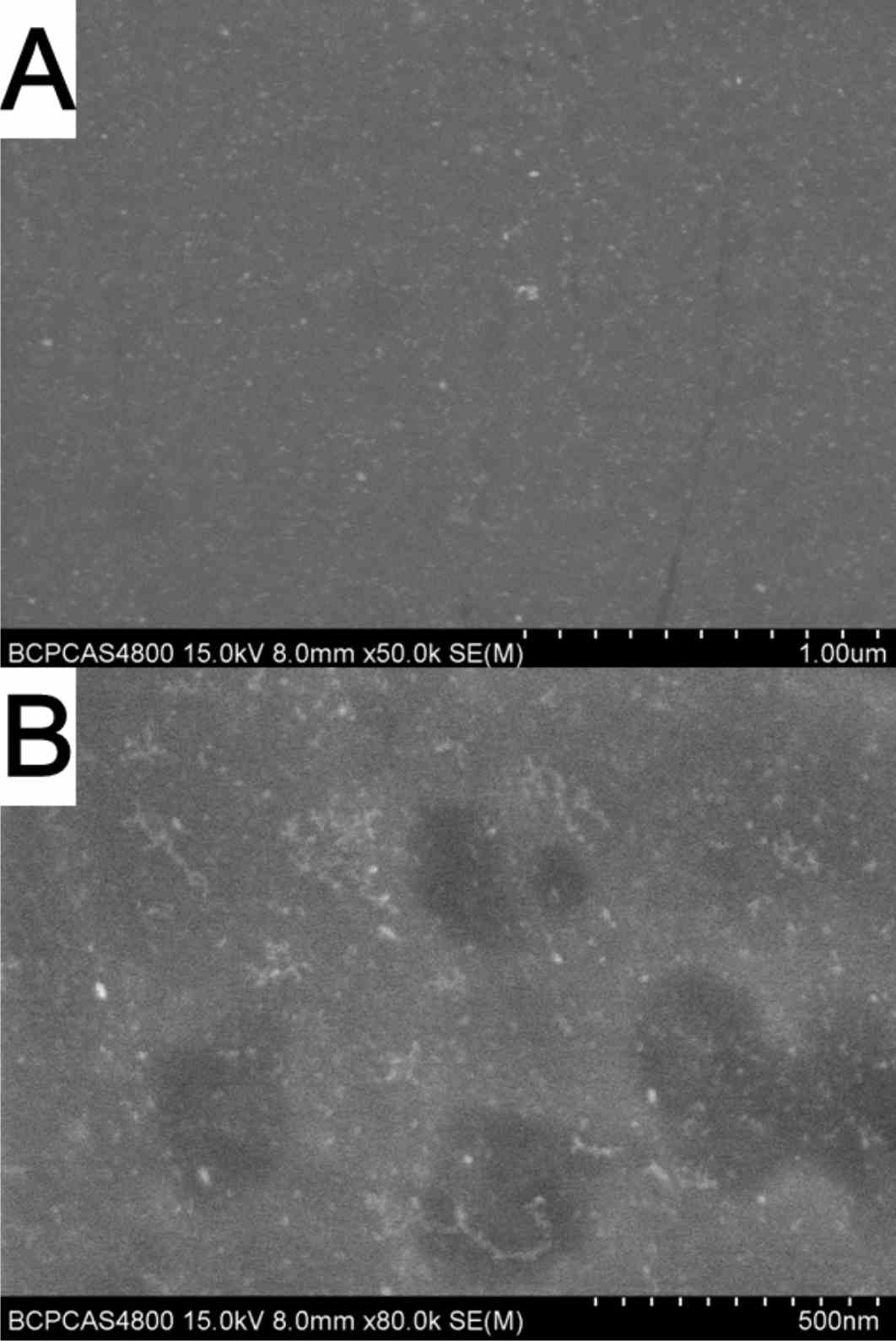


**Figure S1.** SEM images of CMC-Au/GCE (**A**) and CMC-Au-Pb^2+^/GCE (**B**).


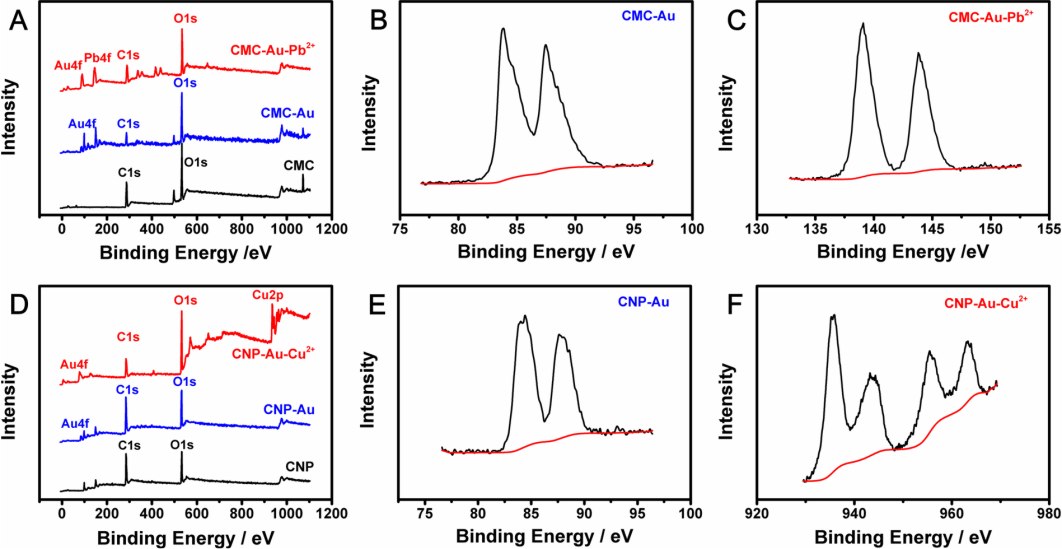


**Figure S2.** (**A**) XPS survey spectra of CMC, CMC-Au and CMC-Au-Pb^2+^. High-resolution XPS spectra of Au4f (**B**) of CMC-Au and Pb4f (**C**) of CMC-Au-Pb^2+^. (**D**) XPS survey spectra of CNP, CNP-Au and C-Au-Cu^2+^. High-resolution XPS spectra of Au4f (**E**) of CNP-Au and Cu2p (**F**) of C-Au-Cu^2+^.


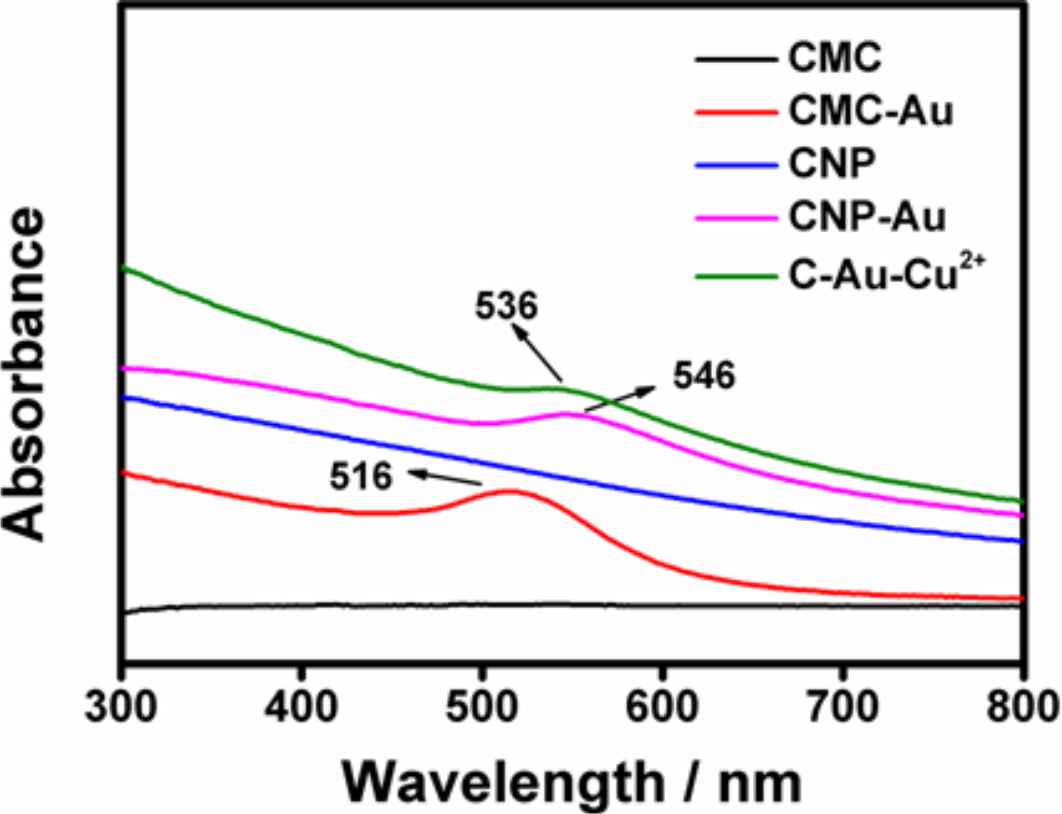


**Figure S3.** UV-vis spectra of CMC, CNP, CMC-Au, CNP-Au, CMC-Au-Pb^2+^ and C-Au-Cu^2+^.


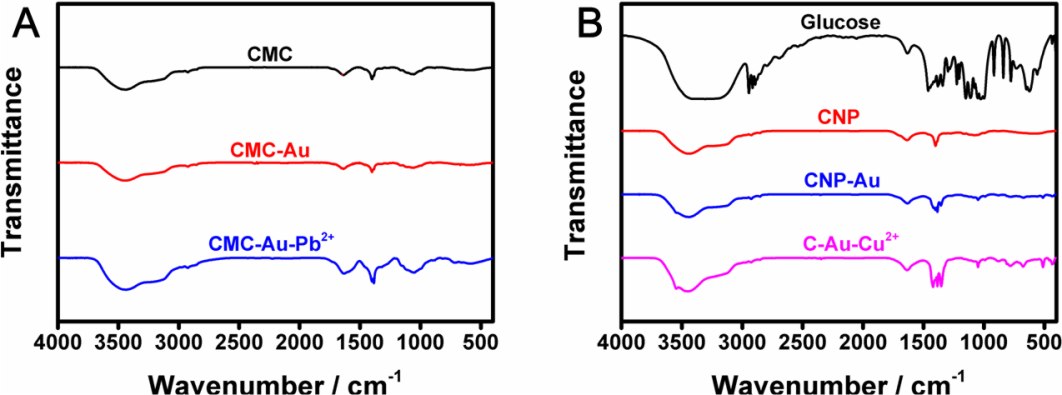


**Figure S4.** (**A**) FT-IR spectra of CMC, CMC-Au and CMC-Au-Pb^2+^ CNP. (**B**) FT-IR spectra of CNP-Au and C-Au-Cu^2+^.

**
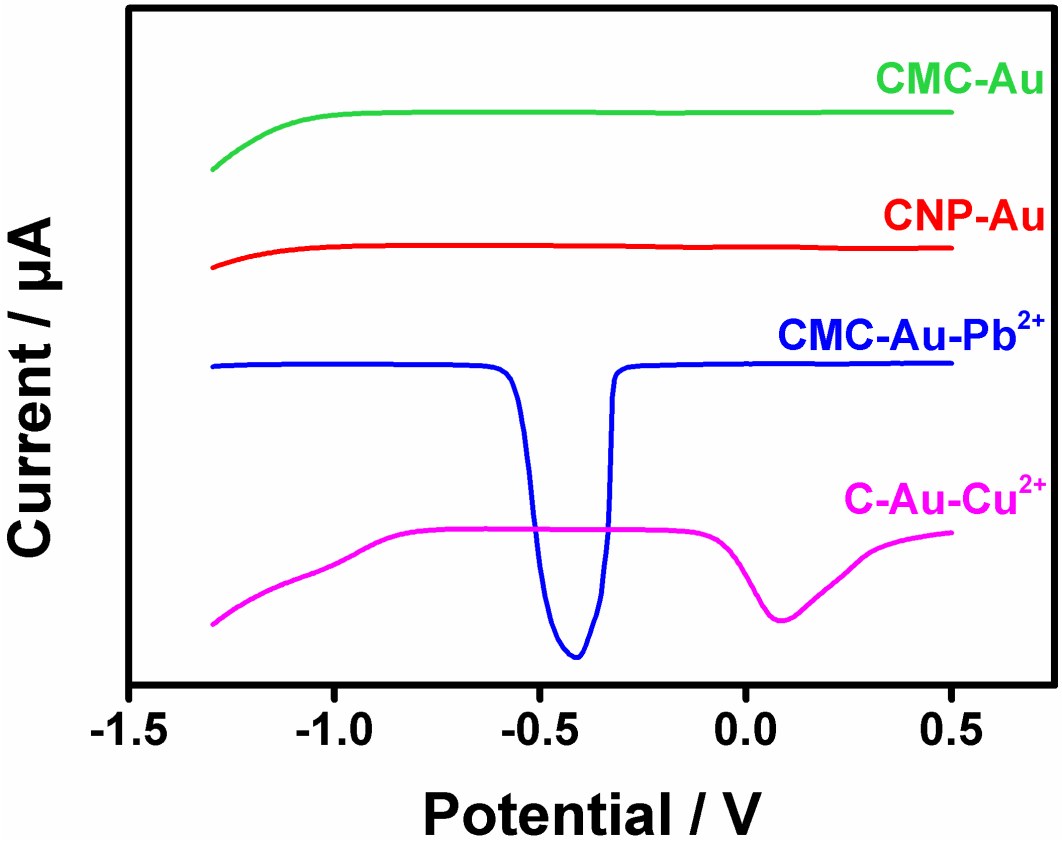
**

**Figure S5.** SWV responses of different electrodes treated with: CMC-Au, CNP-Au, CMC-Au-Pb^2+^ and C-Au-Cu^2+^, respectively.


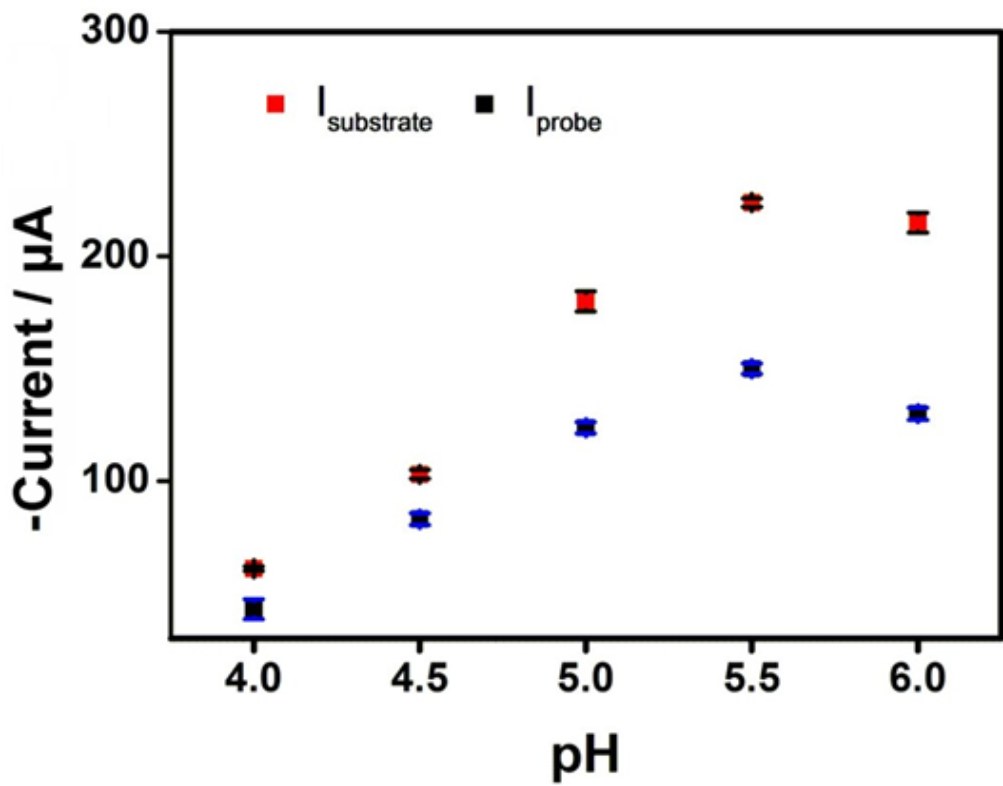


**Figure S6.** Effect of the pH of detection solution on amperometric response of the proposed immunosensor.


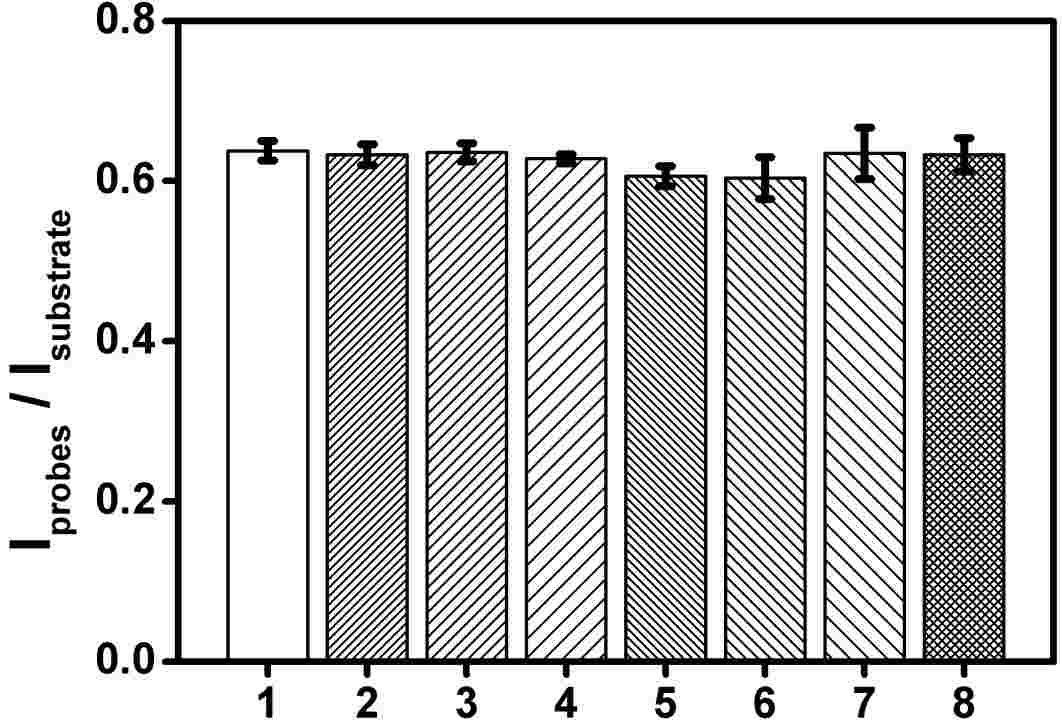


**Figure S7.** Current responses of the proposed immunosensors toward: 10 ng mL^-1^ IgG (**1**), 10 ng mL^-1^ IgG + 100 ng mL^-1^ GC (**2**), 10 ng mL^-1^ IgG + 100 ng mL^-1^ DA (**3**), 10 ng mL^-1^ IgG + 100 ng mL^-1^ UA (**4**), 10 ng mL^-1^ IgG + 100 ng mL^-1^ AA (**5**), 10 ng mL^-1^ IgG + 100 ng mL^-1^ Glu (**6**), 10 ng mL^-1^ IgG + 100 ng mL^-1^ LA (**7**), 10 ng mL^-1^ IgG + 100 ng mL^-1^ BSA (**8**).

**Table S1.** Determination of IgG in human serum samples with the proposed immunoassay and ELISA method.

| **Sample no.** | **ELISA** | **This work** | **RE** |
| --- | --- | --- | --- |
|  | (ng mL^-1^) | (ng mL^-1^) | (%) |
| 1 | 1.03 | 1.00 | -4.5 |
| 2 | 1.17 | 1.22 | 4.3 |
| 3 | 0.73 | 0.70 | -4.1 |
| 4 | 1.17 | 1.22 | 4.5 |
| 5 | 1.53 | 1.56 | 2.2 |
| 6 | 0.56 | 0.55 | -0.9 |
| 7 | 1.33 | 1.32 | -1.0 |
| 8 | 1.45 | 1.36 | -5.9 |
| 9 | 0.98 | 1.03 | 5.3 |
| 10 | 1.61 | 1.63 | 1.2 |
